# The Use of Hammett Constants to Understand the Non-Covalent Binding of Aromatics

**DOI:** 10.5936/csbj.201204004

**Published:** 2012-03-06

**Authors:** Michael Lewis, Christina Bagwill, Laura K. E. Hardebeck, Selina Wireduaah

**Affiliations:** a Saint Louis University, 3501 Laclede Avenue, Saint Louis, Missouri, USA 63130

**Keywords:** Hammett Constants, Arene-Arene Interactions, Cation-Arene Interactions, Aromatics

## Abstract

Non-covalent interactions of aromatics are important in a wide range of chemical and biological applications. The past two decades have seen numerous reports of arene-arene binding being understood in terms Hammett substituent constants, and similar analyses have recently been extended to cation-arene and anion-arene binding. It is not immediately clear why electrostatic Hammett parameters should work so well in predicting the binding for all three interactions, given that different intermolecular forces dominate each interaction. This review explores such anomalies, and summarizes how Hammett substituent constants have been employed to understand the non-covalent binding in arene-arene, cation-arene and anion-arene interactions.

## 1. Introduction

Non-covalent interactions of aromatic π-electron density have been extensively studied [1]. There is a rich history of work aimed at understanding the nature of arene-arene [1, 2] and cation-arene [1, 3] interactions, and the literature contains numerous examples of the importance of these non-covalent complexes in chemistry and biology [1]. For instance, noncovalent interactions of arene rings are important in enzyme-substrate recognition [4], protein folding [5], ion-transport [6], DNA/RNA base-stacking [7], and intercalation [8]. Over the past 10 – 15 years the field of anion-arene binding has received considerable attention [9, 10, 11, 12], and there is increasing evidence the interaction is important in various areas of chemistry [13] and biology [14]. To varying degrees, non-covalent interactions of aromatics with other aromatics, with cations, and with anions have been understood in terms of Hammett substituent constants. Of these three general types of aromatic non-covalent interactions, studies of arene-arene interactions were the first to employ Hammett constants as a means of understanding the binding, generally in the context of experimental physical organic investigations [15]. More recently, cation-arene [16] and anion-arene [17] binding of substituted aromatics have also been described in terms of the relationship with Hammett substituent constants. Although numerous reviews have been written about arene-arene [1, 2], cation-arene [1, 3], and anion-arene [1, 9, 11] interactions, including a very recently published general review of the binding of aromatic π-electron density by Diederich and coworkers [1], there are no reviews that concentrate on the expanding body of work reporting the relationship between the non-covalent binding of aromatics and Hammett substituent constants. Thus, the topic is reviewed here.

## 2. Brief Overview of Hammett Substituent Constants

**Figure 1:**
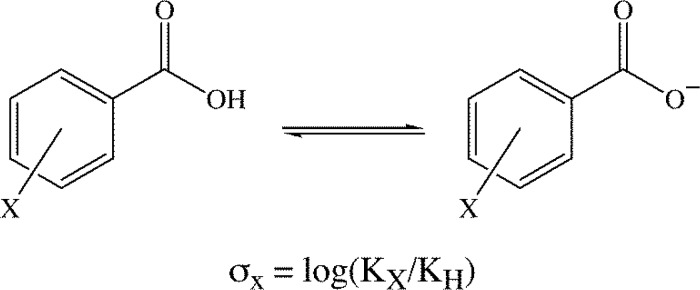
Hammett constants are determined by measuring the effect of substitution on the acid dissociation constants of substituted benzoic acids.

Given the nature of this review, it seems appropriate to give a brief overview of how Hammett substituent constants are derived, and to provide a couple examples of the types of chemical problems they were initially intended to help solve. Hammett constants [18] are determined as shown in Figure 1, and thus explicitly describe the effects aromatic substitution has on benzoic acid ionization [19]. Hammett constants derived from placing the substituent *meta* to the carboxylic acid functional group are termed σ_m_, and are generally recognized as describing the movement of electrons via the σ-framework (inductive effects). The σ_p_ Hammett constant is obtained from substitution *para* to the –CO_2_H group, and it describes the movement of electrons via the σ- and π-framework (inductive and resonance effects). Hammett constants were developed to help explain trends in the reactivity of *meta*- and *para*-substituted benzoic acid derivatives and related compounds. For instance, the electrophilicity of *meta*- and *para*-substituted benzoic esters, the nucleophilicity of *meta*- and *para*-substituted anilines, and the solvolysis of *meta*- and *para*-substituted benzyl halides [19]. Thus, Hammett constants were developed, and initially employed, to describe the reactivity at an atom directly bonded to an aromatic, *meta*- or *para*- to a substituent. It is not clear to the authors why such a parameter should correlate with the non-covalent binding energies of substituted aromatics. Even more curious is the fact that the non-covalent binding energies of interactions as different as arene-substituted arene, cation-substituted arene, and anion-substituted arene interactions have all been shown to correlate with Hammett substituent constants. A brief commentary on this seemingly puzzling body of work is given after reviewing the subject.

## 3. Arene-Arene Interactions

**Figure 2:**
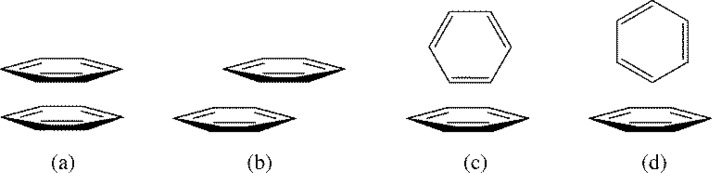
Common benzene-benzene dimer conformations: (a) parallel face-to-face; (b) parallel offset; (c) edge-to-face; (d) T-shaped.

**Figure 3:**
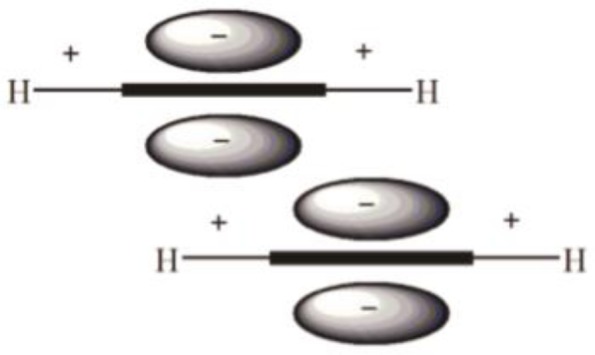
Proposed electrostatic attraction between two benzene rings in a parallel offset conformation. The positively charged σ-framework is attracted to the negatively charged π-electron density.

Experimental observations of arene-arene interactions have been noted for quite some time, notably in DNA/RNA base pair stacking, protein folding and structure, and many other chemically and biologically relevant examples [1], yet the specific nature of this interaction on a molecular level has yet to be fully understood. It is clear, however, that the interaction between two aromatics is a complex phenomenon involving the interplay of various forces contributing to an overall attractive interaction. In an early and broad look at arene-arene interactions, Hunter and Sanders described the interaction energy between two aromatic systems as being comprised of four terms: electrostatic, induction, dispersion, and repulsion [20]. Together, the contribution of each component was thought to adequately account for experimental observations of π-π interactions. While an attractive interaction between two negative π densities of stacked aromatics is seemingly counterintuitive, Hunter and Sanders rationalized a favourable arene-arene interaction as being the result of the positively charged σ-framework of one aromatic interacting with the negatively charged π-electron density of the other aromatic [20]. Figure 2 shows four standard conformations for benzene-benzene dimers, and the Hunter-Sanders model explains why the parallel offset (Figure 2b), the edge-to-face (Figure 2c), or the T-shaped (Figure 2d) geometries are more stable than the parallel face-to-face geometry (Figure 2a). The σ-π attraction model suggested by Hunter and Sanders is illustrated in Figure 3 to explain the attraction between two benzenes in the parallel offset conformation (Figure 2b).

Consistent with the view that multiple factors contribute to the overall attraction between two aromatics, Hunter and Sanders made a distinction between the role of each term, suggesting the electrostatic term controls the geometric preference of the arene-arene system while a majority of the energetic contribution arises from the other terms [20]. If the conformational preference of two interacting aromatics is driven by electrostatics, it would be reasonable to expect the interaction energies would be related to Hammett substituent constants, which capture, in part, the inductive and through-space electrostatic capabilities of substituents. However, at the time Hunter and Sanders published their work, the explicit use of the Hammett constant to describe the interaction of aromatics had sparsely been used. An early example of the use of Hammett constants to describe aromatic interactions from Nicolas and coworkers, though not arene-arene interactions, showed that for 4-substituted arene-carboxylate interactions there was a correlative relationship between carbonyl ^13^C shifts and the σ_p_ for the 4-substituted arene [21].

**Figure 4:**
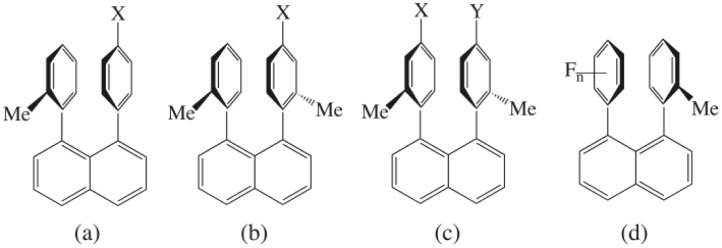
1,8-diarylnaphthalene systems studied by Cozzi, Siegel and co-workers to investigate parallel face-to-face arene-arene interactions: (a) and (b) mono-substituted systems; (c) disubstituted systems; (d) fluorinated benzene-substituted benzene systems [15, 22, 23].

Similar to the notion put forth by Hunter and Sanders that the interaction energy of aromatics can be divided into various terms, Cozzi, Siegel and coworkers presented a further simplified division of the interaction energy where coulombic (electrostatic) and van der Waals (dispersion) terms were thought to be the main contributing components to arene-arene interactions [15]. A dependence of van der Waals interactions on surface area was emphasized and in accordance with this assumption, the dispersion term was thought to be negligible due to the limited surface area of a benzene molecule [15]. To probe these ideas, Cozzi, Siegel and co-workers synthesized a series of 1,8-diarylnaphthalenes with the general structures shown in Figure 4a and 4b to investigate the interaction between two aryl groups in a parallel face-to-face conformation [15]. The barrier of rotation, ΔG^‡^, of the aryl groups was measured and substituent effects were observed. Substitution of an aromatic hydrogen atom with an electron-donating group (Figure 4a and 4b), such as an alkyl, methoxy, or amino group, was predicted to induce an increasingly unfavourable effect due to repulsion of the increasingly negative electron densities forming on the aromatic centre [15]. Conversely, the addition of an electron-withdrawing group (Figure 4a and 4b), such as a halogen, nitro, or cyano group, was thought to induce an increasingly favourable interaction due to the decrease of negative electron density on the aromatic centre. This general view of substituent effects on arene-arene interactions presented by Cozzi, Siegel and coworkers has proven to be an important reoccurring concept that has been the subject of further study. Hammett constants possess information about an aromatic substituent's ability to donate to or accept electron density from an aromatic centre, and thus they were used to understand the rotational barrier of the substituted 1,8-diarylnapthalenes (Figure 4) [15]. A general trend was observed that as the electron withdrawing ability of a substituent increased, the rotational barrier also increased due to the stabilization of the ground state of the molecule by the reduction of repulsive forces, and this correlated quite well with the Hammett σ_p_ value [15]. This correlation led the authors to conclude that a through-space interaction, coulombic (electrostatic) in nature, was occurring between the two aryl groups [15].

Cozzi, Siegel and coworkers expanded their work to 1,8-diarylnaphthalenes with the general structure shown in Figure 4c to investigate charge-transfer effects [22]. The barrier to epimerization of substituted *syn* and *anti* 1,8-di-o-tolylnaphthalenes where each tolyl group was substituted para to the naphthyl ring (Figure 4c) was studied, and the observed trends were the same as in their previous study. Electron-withdrawing substituents stabilize the ground state due the reduction of repulsive forces, and this increases the barrier to rotation. Once again, a correlation was found between the rotational barrier and the Hammett substituent constants, this time the sum of the Hammett constants for the two substituents (Σσ_p_), and this led to the conclusion that charge-transfer effects should be expected to minimally contribute to the overall aromatic interaction as compared to the electrostatic contribution [22]. This work suggests an additivity rule for Hammett constants, where interactions between aromatics involving multiple substituents correlate with the sum of the Hammett constants. Cozzi and Siegel further explored this issue with the rotational barrier of 1,8-diarylnaphthalenes where the fluorination of one of the aromatics is increased from 1 to 5 (Figure 4d) [23]. It was hypothesized that with each additional fluorine atom, the aromatic core would become less electron-rich, leading to an increase in the attraction with the neighbouring aromatic and an increased barrier to rotation. This is indeed what was observed, and the measured ΔG^‡^ values correlate well with the sum of the fluorine Hammett constant (Figure 4d) [23].

**Figure 5:**
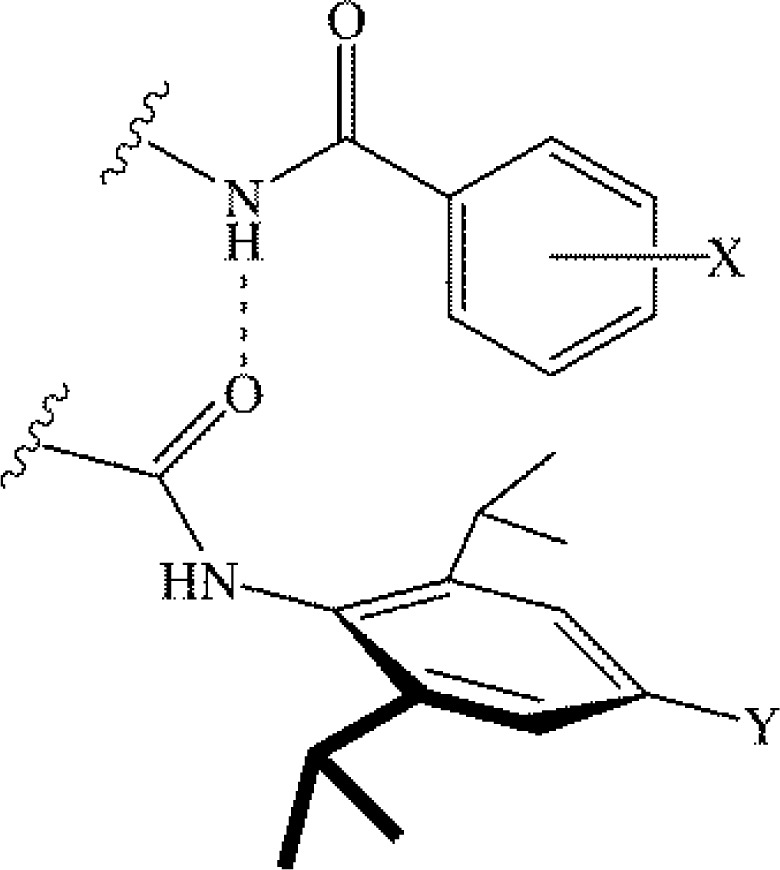
Edge-to-face aromatic interaction investigated by Hunter and coworkers via chemical double-mutant cycles. The Y-substituted ring (Y = NMe_2_, H, NO_2_) is considered the face ring. The *meta*- or *para*-X-substituted ring, (X = NMe_2_, H, t-Bu, or NO_2_) is considered the edge ring [24].

The Cozzi, Siegel and coworkers studies recognized the significant, and seemingly dominant, contribution that electrostatics have in arene-arene interaction energies, specifically for parallel face-to-face interactions. One of the primary results supporting this interpretation was the correlation between the barriers to rotation and the Hammett substituent constants, or the sum of the Hammett constants. The prevailing notion that electrostatics could dominate arene-arene interactions without significant competition from other forces, such as dispersion, was examined for edge-to-face arene-arene interactions by Hunter and coworkers using their chemical double-mutant cycles [24]. In the systems studied the face ring is substituted with Y = NMe_2_, H, and NO_2_ to capture the effects of substituting with an electron donating, neutral, and electron withdrawing group, respectively (Figure. 5). The edge ring was substituted in both the *meta*- and *para*-position with X = NMe_2_, H, and NO_2_ and t-Bu in the *para*-position only (Figure 5) [24]. The observed binding trends were largely explained via electrostatic arguments; for instance, when both rings were substituted with a nitro group, and were thus electron deficient, the interaction was unfavourable. For variations in the Y group and in the *meta*-X group, there was a correlation between the interaction energy and the Hammett σ_p_ parameter; however, no correlation existed for variations in the *para*-X substituent (Figure 5) [24]. Ultimately, it was concluded that electrostatics accounted for the changes in interaction energy because of the reasonable correlations with Hammett substituent constants [24].

In addition to Hammett constants being used to understand the binding in parallel face-to-face (Figure 2a) and edge-to-face (Figure 2c) arene-arene interactions, they have also been employed in studies of parallel offset arene-arene binding. Cozzi, Siegel and coworkers extended previous studies that had correlated the rotational barrier of parallel face-to-face aryl groups with σ_p_ [15] and Σσ_p_ [22] by investigating a new series of compounds where a parallel offset conformation of aromatics is observed [25]. In these compounds, a rotating substituted phenyl group partially overlaps another aromatic unit in a conformationally constrained polycyclic system. It was predicted that substituent effects would be less pronounced due to the incomplete overlap of π-electron density of the two aromatic groups, which would decrease repulsive tendencies [25]. Consistent with this prediction, the rotational barrier in this system was less than the rotational barrier of previously studied 1,8-diarylnaphthalenes [25]. Despite this, the same trends were observed for the parallel offset systems as for the parallel face-to-face interactions: substitution with an electron withdrawing group lowered the energy of the ground state molecule and thus increased the rotational barrier and the opposite trend was observed with electron donating groups. Furthermore, there was an excellent correlation between the measured rotational barrier and the substituent σ_p_ values [25]. Due to the displacement of an aromatic group in the parallel offset conformation, the possibility arises for an attractive interaction between a hydrogen atom of one aromatic and the π-electron density of the other, as described by Hunter and Sanders [20]. Cozzi, Siegel and coworkers, however, concluded that this interaction would not be a significant contributor to the overall binding, and that electrostatics are the dominant factor in parallel arene-arene interactions, due to correlation of both parallel face-to-face and parallel offset binding energies with Hammett substituent constants [25].

**Figure 6:**
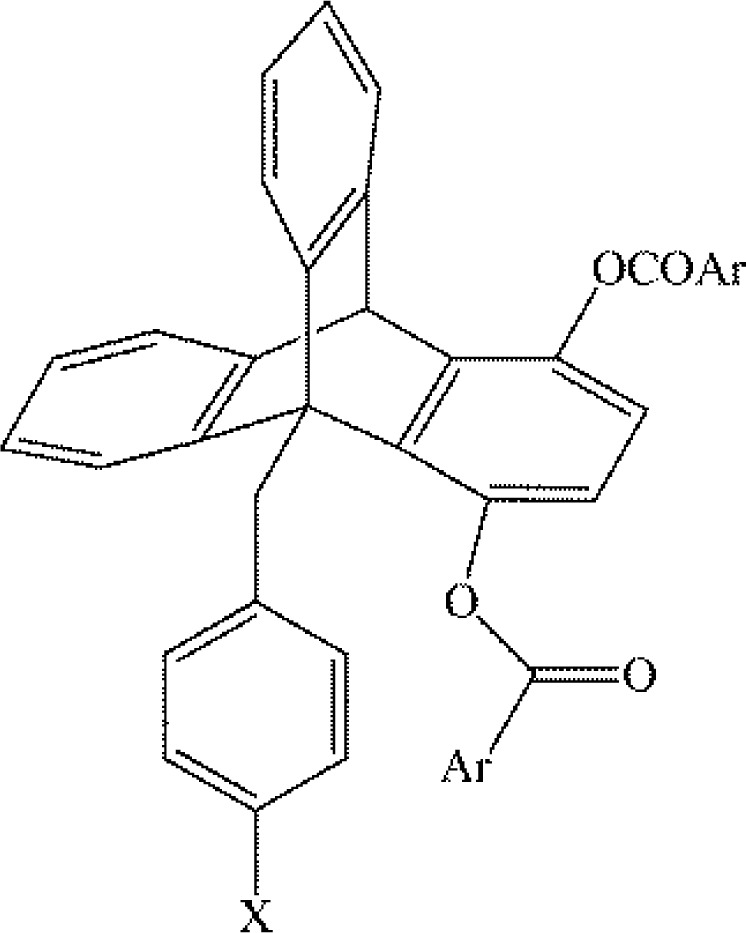
1,9-diaryl-substituted triptycene molecules studied by Gung and coworkers. The aryl groups (Ar) are varyingly electron deficient through substitution with electron withdrawing groups. The substituents (X) on the benzyl group vary from electron donating to electron withdrawing. The interacting aromatic units assume a parallel offset conformation [26].

The absence of charge-transfer absorption bands from UV-Vis spectra had been the primary evidence against the inclusion of charge-transfer effects in the study of arene-arene binding. Gung and coworkers probed the parameters of when it was appropriate to include, or exclude, charge-transfer effects in parallel offset arene-arene interactions through the investigation of 1,9-diaryl-substituted triptycene systems (Figure 6). Triptycenes were studied where the two aromatics are in a parallel offset conformation, and where one of the aromatics was strongly electronically perturbed, such as 4-nitrobenzoate and perfluorobenzoate (Figure 6) [26]. When one aromatic was held constant as 4-nitrobenzoate (Figure 6 where Ar = 4-C_6_H_4_(NO_2_), and the adjacent aromatic was mono-substituted at the 4-position with various substituents (Figure 6 where X = N(CH_3_)_2_, OCH_3_, CH_3_, H, F, CF_3_), the effect of substitution were in accordance with previous studies (i.e. a stronger electron withdrawing group leads to a more attractive interaction) and there was a strong correlation with the Hammett constant σ_p_ [26]. When the electron deficient group was changed to Ar = C_6_F_5_ the linear correlation with σ_p_ significantly deteriorated. Interestingly, the series (Figure 6, Ar = C_6_F_5_) that showed a deviation from linearity between the arene-arene binding energy and the Hammett σ_p_ value had UV-Vis charge-transfer bands for the triptycene analogs where the X-substituted aromatic had electron-donating substituents (Figure 6) [26]. Thus, for arene-arene systems where one aromatic is strongly electron deficient and the other aromatic is electron rich, factors other than electrostatics must be considered, as evidenced by the lack of correlation between the arene-arene binding energies and the Hammett σ_p_ parameter [26]. To supplement their intriguing experimental results of fluorinated aromatics, Gung and Amicangelo initiated an extensive theoretical study of perfluorobenzene-substituted benzene dimer systems where calculations were performed on parallel offset and parallel face-to-face arrangements [27]. The results from this study mirrored the experimental results; a non-linear correlation between the binding energies and σ_p_ was observed due to a higher than normal binding energy between the perfluoro-aromatics and electron-rich aromatics. These results were again used to conclude that charge-transfer effects contribute to arene-arene binding when electron-poor and electron-rich aromatics interact [27].

Computational studies by Sherrill and coworkers offered further deviation from the electrostatic model of arene-arene binding, where the Hammett substituent constant alone was not sufficient to predict T-shaped benzene-substituted benzene binding energies [28]. Symmetry adapted perturbation theory (SAPT) calculations indicated a combination of dispersion and electrostatics were the most significant factors in the overall interaction energy, and this led Sherrill and coworkers to propose a multi-parameter model to describe the binding in T-shaped benzene-substituted benzene binding [28]. The Σσ_m_ value was employed to describe electrostatics, a polarizability parameter was used to take into account the effects of dispersion, and a parameter to describe direct interactions between the substituents of the substituted benzene and the H-atoms of the benzene was included [28]. In addition to the use of a multi-parameter equation to describe arene-arene binding, the work by Sherrill and coworkers also deviated from most previous studies in employing the Hammett σ_m_ value, rather than the σ_p_ value, to describe the effects of electrostatics.

Hunter and coworkers employed their chemical double-mutant cycles to investigate parallel offset arene-arene binding and found an indirect correlation between the experimentally measured binding energies and the Hammett σ_m_ value [29]. The work involved a large number of substituted arene-substituted arene interactions, and when one aromatic was held constant as either the perfluoro-analog or the 2,6-dimethyl-analog, while the adjacent aryl group was variously substituted, the binding energies correlated quite well with the B3LYP/6-31G* calculated electrostatic potential (ESP) of the substituted aryl group. Dougherty and coworkers had previously shown that ESP values of substituted aromatics correlated very well with Hammett σ_m_ values [30], and Hunter and coworkers showed this correlation held for the aromatics investigated in their studies [29]. Thus, the indirect correlation between the arene-arene binding energies and Hammett σ_m_ values.

**Figure 7:**
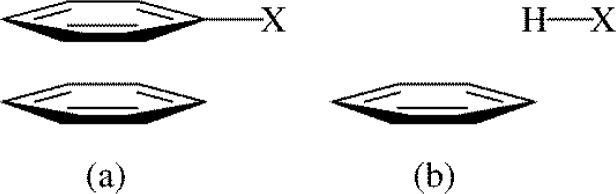
Houk and Wheeler model for the origin of substituent effects: the binding energy of substituted benzene-benzene dimers (a) can be approximated via the binding energy of HX-benzene dimers (b) where X is the substituent in the substituted aromatic [31].

Computational work by Houk and Wheeler suggested a correlation between parallel face-to-face mono-substituted benzene-benzene binding energies and the Hammett σ_m_ value of the substituted benzenes [31]. A correlation with σ_m_ was also observed when perfluorobenzene was substituted for benzene. This work also proposed a simple model for mono-substituted benzene-benzene dimers, and mono-substituted benzene-perfluorobenzene dimers, where the binding energy could be predicted from the benzene-HX or perfluorobenzene-HX binding energy, respectively (Figure 7) [31]. The X group is the substituent from the substituted benzene. The primary evidence for this simplified model was the correlation between the Hammett σ_m_ value and the mono-substituted benzene-benzene, or mono-substituted benzene-perfluorobenzene, binding energy [31]. The excellent correlation between benzene-HX or perfluorobenzene-HX binding energies and the Hammett σ_m_ value led Houk and Wheeler to propose the importance of substituent-arene interactions in arene-arene binding.

Houk and Wheeler expanded the model in Figure 7 to edge-to-face arene-arene interactions [32]. The model isn't quite as easy to apply as it was for the parallel face-to-face arene-arene system, as the nature of the benzene-HX dimer depends on whether the edge ring or face ring is substituted in the edge-to-face dimer [32]. If the edge ring is substituted, the benzene-HX dimer is essentially identical to what is shown if Figure 7b. If the face ring is substituted, the benzene-HX dimer has the HX interacting with the σ-framework of the benzene. Ultimately, the results were not nearly as clean as for the parallel face-to-face work. First of all, when the edge ring is substituted, the correlation between the substituted benzene-benzene binding energy and the Hammett σ_m_ value is excellent, but when the face ring is substituted the correlation with σ_m_ is not very good [32]. Second, the benzene-HX model works well in predicting the edge-to-face substituted benzene-benzene binding energy when the face ring is substituted, however when the edge ring is substituted the benzene-HX model doesn't work as well. Ultimately, Houk and Wheeler conclude that the edge-to-face arene-arene binding energies were dictated by direct interactions of the substituents with the other aromatic, as well as electrostatic interactions of an H atom of the substituted ring with the π cloud of the unsubstituted ring [32].

Recent work by Gung and coworkers challenged the importance of substituent-arene interactions in arene-arene binding by employing a triptycene model systems similar to the one shown in Figure 6, except the aryl group Ar is replaced with a C(O)CH_2_X group [33]. This model resulted in CH-substituted benzene binding, and regardless of whether the substituted aromatic was held constant and the substituted ester was varied, or the substituted ester was held constant and the substituted aromatic was varied, the binding energy correlated very well with the σ_m_ value of either the substituted ester or the substituted aromatic. This led to Gung and coworkers suggesting the Houk and Wheeler model illustrated a π-H -bond interaction and not a substituent-arene interaction.

Wheeler has very recently reported more work supporting the importance of substituent-arene interactions in arene-arene binding [34]; however the work does not involve correlations with Hammett constants. Given the focus of this review, the more important response to Houk's and Wheeler's initial study of parallel face-to-face mono-substituted benzene-benzene binding comes from Sherrill and Ringer where they showed that when multi-substituted benzenes, and benzenes with electron-donating substituents, were considered, there is in fact no correlation between parallel face-to-face arene-arene binding energies and Hammett Σσ_m_ values [35]. The sum of the Hammett constants were used since multi-substituted benzene were investigated. The Houk and Wheeler study included primarily benzenes with electron-withdrawing substituents and, as has been noted, included only mono-substituted benzenes [31]. Two previous studies by Sherrill and coworkers showed that (i) compared to the benzene-benzene binding energy, adding any substituent, electron-donating or electron-withdrawing, results in a more stable parallel face-to-face substituted benzene-benzene binding energy [36]; (ii) adding more substituents, electron-donating or electron-withdrawing, results in a more stable parallel face-to-face substituted benzene-benzene binding energy [28]. As Sherrill and Ringer noted and demonstrated [35], these results are incompatible with a correlation between parallel face-to-face arene-arene binding energies and Hammett substituent constants or, for that matter, any electrostatic parameter. Sherrill and coworkers have performed SAPT calculations that demonstrate the importance of dispersion in parallel face-to-face arene-arene binding, and the lack of correlation between the binding energy and Hammett substituent constants supports this finding.

Recent work by Lewis and coworkers reported an extensive computational study of parallel face-to-face substituted benzene-benzene binding where mono-substituted and multi-substituted benzenes with a wide range of electron donating and withdrawing capabilities were investigated [37]. This expanded on previous work showing a correlation between parallel face-to-face arene-arene binding energies and Σσ_p_ values [38]. As was the case in the Sherrill and Ringer study, there was no correlation between the binding energy and the Σσ_m_ value; however, the correlation with the Σ∣σ_m_∣ value was quite good [37]. SAPT calculations revealed that the energy due to electrostatics varied significantly, correlating to a decent degree with the Σ∣σ_m_∣ value, and the combined energy due to dispersion, induction and exchange is relatively constant [37]. This helped explain why electrostatic parameters, like Hammett constants, have proven so successful in correlating with arene-arene binding energies. Furthermore, as had been shown by Sherrill and coworkers [28, 36], the Lewis and coworkers study showed that dispersion is the dominant contributor to the overall binding energy [37]. Although it remains largely unclear why the parallel face-to-face binding energies correlated with Σ∣σ_m_∣ values, or what a ∣σ_m_∣ value even means, the significant variation in the energy due to electrostatics coupled with the dominance of the energy due to dispersion led Lewis and coworkers to propose a two-parameter model for predicting parallel face-to-face substituted benzene-benzene binding energies. Using the Hammett Σσ_m_ term to describe electrostatics and the sum of the molar refractivity constant M_r_ (ΣM_r_) to describe dispersion led to an excellent correlation between the calculated and predicted binding energies. This led to Lewis and coworkers suggesting the ∣σ_m_∣ value contain information about the electrostatic and dispersion/polarizability properties of a substituent, though this initial hypothesis should be tested more vigorously.

Before moving on from the use of Hammett constants to understand arene-arene interactions, it seems important to note a discrepancy between the views of the experimentalists and the computational researchers. In general, the former have tended to note the correlation between arene-arene binding energies and Hammett constants, while the latter have found Hammett constants inadequate to predict arene-arene binding energies. Computational researchers have largely cited the importance of dispersion in arene-arene interactions as a primary factor for the shortcomings of relying solely on Hammett values. However, Hunter, Cockroft and coworkers have noted the important point that the default for *ab initio* calculations is the gas-phase and, as a result, they suggest the importance of dispersion in the gas-phase arene-arene interactions is due to the lack of a desolvation term [29, 39]. Solution-phase computational work would help address this important issue.

## 4. Cation-Arene Interactions

**Figure 8:**
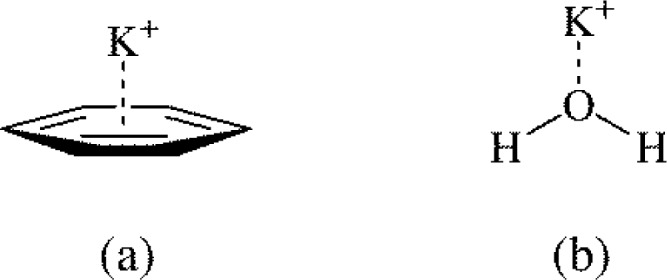
The general cation-π structure (a) has a cation over the center of the aromatic π-cloud, and is as strong as cation-water binding (b) in the gas-phase [40].

Like arene-arene interactions, there is a relatively long history of research on cation-π interactions of aromatics [1, 3]. Cation-π interactions have the cation over the centre of the aromatic π-density (Figure 8a), and the seminal work of Kebarle and coworkers showed that K^+^-benzene binding (Figure 8a) was as strong as K^+^-water binding (Figure 8b) in the gas-phase [40]. The work of Burley and Petsko suggested the importance of cation-π interactions in protein stability [41], and Dougherty and coworkers have published numerous important studies investigating the nature of cation-π interactions and the importance in various biological fields [3, 42]. The nature of the cation-π interaction has largely been discussed in terms of electrostatics [3], though it has also been suggested that π-cloud induction [43, 44] and cation-substituent interactions [45] may play a role in the binding. The term most commonly employed to understand cation-π binding has been the aromatic quadrupole moment, Θ_zz_ [3]. The aromatic Θ_zz_ value has been shown to correlate to a decent degree with the cation binding of substituted benzenes [46], and it has been used to explain the differential solid-state K^+^ binding ability of certain substituted aromatics [47]. Although there is not nearly as much work investigating the correlation between cation-π binding energies and Hammett substituent constants as there is for arene-arene interactions, over the past few years the subject has received increased attention and it is reviewed here.

Dougherty and coworkers appear to be the first researchers to suggest a possible relationship between cation-π binding and Hammett substituent constants [30]. In a computational study of the Na^+^ binding of substituted benzenes, pyridine and naphthalene, they suggest the binding of a subset of the aromatics (C_6_H_5_X; X = H, F, OH, NH_2_, BH_2_) roughly correlates with the Hammett constant σ_m_ [30]. This result is interpreted to mean inductive effects are important in cation-π binding, though the remainder of the study concentrates on the correlation between the binding energies and the electrostatic potentials for the entire set of aromatics.

**Figure 9:**
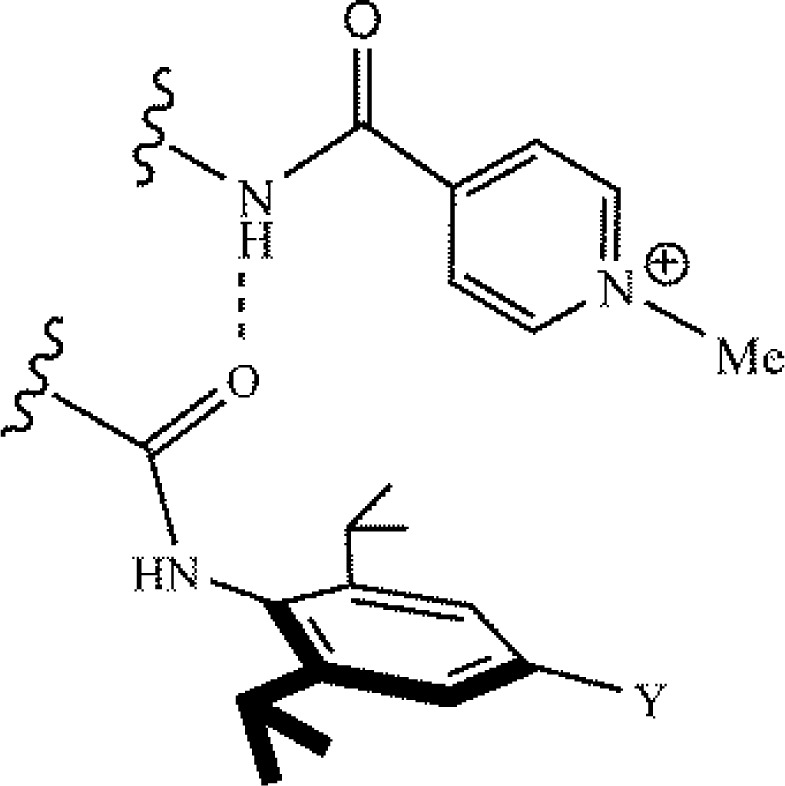
Cation-π interaction investigated by Hunter and coworkers via chemical double-mutant cycles. The Y-substituted group was either NO_2_, H, or NMe_2_ [16].

Hunter and coworkers employed their chemical double-mutant cycles towards the investigation of cation-π binding using the *N*-methyl pyridinium cation [16]. The cation-π complex is shown in Figure 9, and it is similar to the complex Hunter and coworkers employed to study edge-to-face arene-arene binding (Figure 5) [24]. The aromatic was substituted with Y = NO_2_, H and NMe_2_, and there was an excellent correlation between the cation-π binding energies and the Hammett σ_p_ value [16]. Interestingly, the correlation with the Hammett parameter allows for a comparison between the cation-π and edge-to-face arene-arene binding energies, and this shows that the cation-π binding is much more sensitive to changes in the substituent than arene-arene edge-to-face binding [16].

The cation binding of Li^+^, Na^+^, K^+^, Be^2+^, Mg^2+^, and Ca^2+^ with aniline, toluene, phenol, benzene, fluorobenzene, 1,4-difluorobenzene, and 1,3,5-trifluorobenzene was investigated by Jiang and coworkers, and an excellent correlation was found between the binding enthalpies and what the authors term the total Hammett parameter, σ_Total_ [48]. The total Hammett parameter was defined as σ_Total_ = (Σσ_m_ + Σσ_p_). This is the only example of using the σ_Total_ parameter to understand the non-covalent binding or aromatics, and Jiang and coworkers suggest it means both resonance and induction are important in cation-π binding.

**Figure 10:**
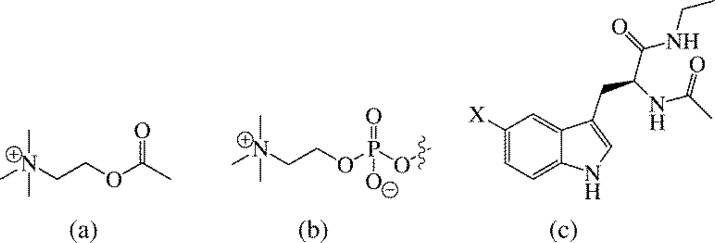
Structure of cationic neurotransmitter acetylcholine (a), the cationic amine 1,2-dimyristoyl-*sn*-glycero-3-phosphocholine (b) and substituted tryptophan analogs (X = OCH_3_, CH_3_, H, F, Cl, Br, I, NO_2_) investigated by Sanderson and coworkers (c) [53].

The binding of neurotransmitters, such as acetylcholine, to the nicotinic acetylcholine receptor has been used to highlight the importance of cation-π interactions in biology [49]. Furthermore, acetylcholine esterase inhibitors have been widely studied as possible treatments for Alzheimer's disease [50, 51]. Since acetylcholine is an ammonium cation (Figure 10a), the binding of cations to aromatic amino acid residues has been an active area of research [52]. Sanderson and coworkers recently reported a very interesting study on the binding of 1,2-dimyristoyl-*sn*-glycero-3-phosphocholine (Figure 10b) to substituted 5-substituted tryptophan analogs (Figure 10c) [53]. The substituents investigated were X = OCH_3_, CH_3_, H, F, Cl, Br, I, and NO_2_ (Figure 10c), and the correlation between the free energy of association and the Hammett constant σ_p_ was far from linear. In fact, a parabolic relationship was observed where the parent tryptophan (Figure 10c, X = H) had the weakest cation binding [53]. Sanderson and coworkers suggested the parabolic relationship between the binding energy and the Hammett σ_p_ value supported contribution from both cation-carbonyl side chain and cation-π interactions to the overall binding energy [53].

Lewis and Cormier recently reported on the correlation between cation-substituted cyclopentadienyl anion (Cp) binding and Hammett substituent constants [54]. Although the nature of cation-Cp binding is very different than the nature of non-covalent cation-π binding of neutral aromatics, the correlation between the binding energies and the Hammett constants are similar, and are thus discussed here. Lewis and Cormier investigated the correlation between the Li^+^-Cp and Na^+^-Cp binding energies and the Θ_zz_, Σσ_p_ and Σσ_m_ values for a large set of mono- and multi-substituted Cp anions. The best correlations were found for the Cp anion Σσ_m_ values; however, if Cp rings with sterically non-hindering groups were considered the correlation with the Cp Θ_zz_ value is quite good [54]. Interestingly, the correlation between cation-Cp binding energies and the Cp Σσ_p_ values is quite poor, thus suggesting that inductive effects are most important for cation-substituted Cp binding [54].

## 5. Anion-Arene Interactions

**Figure 11:**
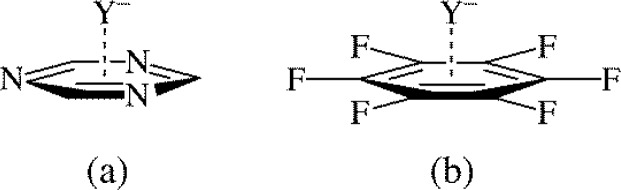
Anion-π interactions between an anion (Y^–^) and the electron deficient aromatics triazine (a) and perfluorobenzene (b).

Anion-π interactions are typically termed as favourable non-covalent interactions between an anion and an electron deficient, π-acidic, aromatic system such as triazine or perfluorobenzene (Figure 11) [1]. Not surprisingly, anion-π interactions of aromatics were largely overlooked as they were expected to exhibit a repulsive interaction between the negatively charged anion and the electron rich area of the aromatic ring [11]. However, three seminal computational studies in 2002 suggested anion-π interactions were attractive [55, 56, 57], and as recent reviews can attest, over the past decade there have been numerous studies supporting the notion that anion-π interactions are attractive [9, 11]. Numerous theories have been offered to explain the nature of the attraction in anion-π interactions. It has been suggested that induction is the dominant force [46] that anion-substituent interactions are important [46, 58], and as expanded on below, correlations between anion-π binding and Hammett substituent constants suggest that electrostatics are important [59]. Still, the field is relatively new, and compared to the more established areas of arene-arene and cation-arene interactions there are fewer studies into the relationship between anion-arene binding and Hammett substituent constants.

**Figure 12:**
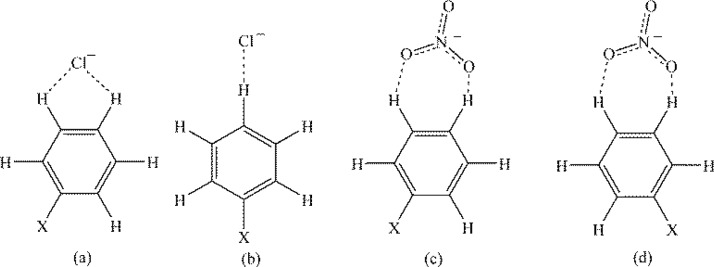
Two possible aryl CH-anion complexes for chloride-substituted benzene complexes (a) and (b) and for nitrate-substituted benzene complexes (c) and (d), studied by Hay and Bryantsev [17].

To the authors’ knowledge Hay and Bryantsev were the first to compare any type of anion-arene interactions to Hammett constants by investigating the influence of substitution on aryl CH-anion hydrogen binding [17]. The computational study included two Cl^–^-substituted benzene complexes (Figure 12a and 12b) and two NO_3_^–^-substituted benzene complexes (Figure 12c and 12d), and the substituted benzene had electron-withdrawing (Figure 12, X = NO_2_, CN, CF_3_, Cl) and electron-donating (Figure 12, X = CH_3_, NH_2_) groups. All complexes were defined with the anion in the plane of the aryl CH and outside of the periphery of the aromatic ring. As would be expected, the presence of electron-donating substituents decreased the binding energy and lengthened the anion-arene distance, and electron-withdrawing substituents increased the binding energy and shortened the anion-arene distance [17]. The strongest binding energy was seen for nitrobenzene and the weakest binding energy was reported for aniline. The resulting binding energies for each complex were plotted against the corresponding substituent's Hammett constant, and the best correlation was achieved with the σ_m_ substituent constant [17]. A poorer relationship was found using σ_p_, and this suggests inductive effects are more relevant that resonance effects [17].

**Figure 13:**
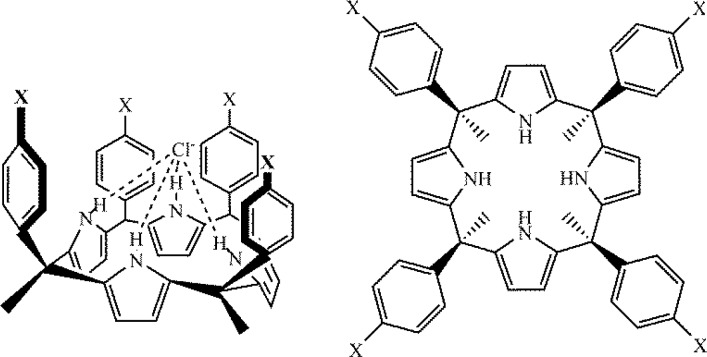
Calix(4)pyrrole receptors employed to investigate the effects of arene substitution on anion-π interactions by Ballester and coworkers [59].

The relationship between anion-π binding energies and Hammett constants was investigated by Ballester and coworkers using substituted analogs of the calix(4)pyrrole system shown in Figure 13, [59]. The chloride binding ability of the calix(4)pyrrole receptor was probed via proton NMR spectroscopy, which showed that the Cl^–^ anion is hydrogen bonded to the four pyrrolic NH groups and experiences anion-π interactions with the attached aromatic groups with apparently little or no occurrence of aromatic hydrogen bonding. The calix(4)pyrrole receptors were tuned by changing the X substituents (Figure 13, X = H, Br, CN, NO_2_, OH, OCH_3_, and OCOCH_3_) [59]. An excellent correlation was found between the difference in the experimentally determined ΔG values and either the Hammett σ_p_ or σ_m_ parameter of the substituent [59]. The correlation with the two Hammett constants was essentially equal, r^2^ = 0.95 for σ_p_ and r^2^ = 0.92 for σ_m_, and thus these results do not provide a clear indication as to whether resonance or induction is dominant in anion-π interactions. Regardless, Ballester and coworkers interpreted the observed trends as supporting an electrostatic interaction between the anion and the aromatic π-density [59].

## 6. Summary and Outlook

As noted in Section 2, Hammett substituent constants were initially developed and employed to understand the reactivity at an atom directly bonded to an aromatic, with substituents in the *meta*- or *para*-positions. As this review details, over the past approximately two decades there has been significant work investigating the correlation between arene-arene binding energies and Hammett substituent constants, and more recently cation-π and anion-π interactions have begun to be studied in this manner. To varying degrees, and depending on the systems studied, the correlations are quite good, and it is not clear to us why this is the case. As we recently stated in our paper on parallel face-to-face arene-arene binding, it is not immediately obvious why a constant that was developed to describe the effects of substitution on the ionization of substituted benzoic acids should correlate to arene-arene non-covalent binding energies [37]. This review allows us to significantly broaden this statement to include all arene-arene interactions, and cation-π and anion-π interactions. It is very difficult to reconcile an electronic parameter, be it σ_m_ or σ_p_, correlating to the binding energy of three non-covalent interactions that are dominated by different forces. Furthermore, within arene-arene, cation-π, and anion-π interactions there is disagreement about what types of forces are important for predicting the binding energies. Arene-substituent [31, 34] and ion-substituent interactions [45, 46] [48] have been touted as being important, induction has been suggested as being important in all three types of interactions [37, 43, 44, 46], and electrostatics have been proposed as the dominant force [1]. The fact that Hammett parameters correlate so well with the binding energies of the three different non-covalent interactions suggests electrostatics is dominant, but the recent work on parallel face-to-face arene-arene binding [31, 35, 37] serves as a warning against such a simple explanation, and further research is certainly warranted. As was the case for parallel face-to-face arene-arene interactions, electrostatics, and Hammett substituent constants, may only be part of the answer on how best to predict the binding energies of arene-arene, cation-π and anion-π interactions.

Of equal interest to discussions about what forces dominate the various interactions, and the role of Hammett constants in predicting the binding energies, is why some studies show the best correlations with σ_m_ or Σσ_m_, while others show σ_p_ or Σσ_p_ to be best. Why would the inductive contributions of a substituent, as measured by σ_m_ or Σσ_m_, sometimes be a better predictor of the binding energies than the combination of the inductive and resonance effects, as measured by σ_p_ or Σσ_p_? Certainly one cannot turn off the resonance withdrawing/donating abilities of a substituent, so why should σ_m_ or Σσ_m_ ever outperform σ_p_ or Σσ_p_? Even more perplexing are the reported correlations with Σ∣σ_m_∣ [37] or σ_Total_, which equals (Σσ_m_ + Σσ_p_) [48]. In the paper discussing the Σ∣σ_m_∣ value the authors suggest the absolute value of ∣σ_m_∣ contains information about the electronic and polarizability properties of a substituent [37], but this is far from definitive. The authors of the work describing σ_Total_ suggest it contains information about both induction and resonance effects, but why is induction counted twice: once in the σ_m_ value and again in the σ_p_ value [48]? Note that the critiques we present here are not meant to be disparaging; we authored the work discussing the Σ∣σ_m_∣ value [37]. We are merely highlighting the fact that many questions remain as to why certain Hammett constants, or permutations of Hammett constants, work best in predicting the binding energies of certain non-covalent interactions of aromatics.

The correlation of Hammett substituent constants with arene-arene, cation-π and anion-π binding energies has allowed researchers to comment on the forces that govern the interactions; however, we believe these studies have produced as many questions as they have addressed. At a few places in this review we have noted that some studies observe the best correlations with σ_m_, while for others it is σ_p_, and for others still it is some manipulation of these parameters such as ∣σ_m_∣ or (σ_m_+σ_p_). In some cases the differences between the cited studies can be attributed to the investigation of different substituted aromatics. Still, this is not always the case, and significant work remains to determine why Hammett constants have performed so well in predicting the binding energies of the interactions discussed here, and why sometimes σ_m_ gives the better correlations, while in other instances σ_p_ performs best. Finally, as noted by Hunter, Cockroft and coworkers, the importance of dispersion in gas-phase interactions may be due to the lack of a desolvation term [29, 39], and thus electrostatics, and Hammett substituent constants, may prove enough to predict solution-phase binding energies, even if they do not predict gas-phase binding energies. As stated above, solution-phase computational work would help address this important issue.
